# LNG-IUS vs. medical treatments for women with heavy menstrual bleeding: A systematic review and meta-analysis

**DOI:** 10.3389/fmed.2022.948709

**Published:** 2022-08-25

**Authors:** Sijing Chen, Jianhong Liu, Shiyi Peng, Ying Zheng

**Affiliations:** Department of Obstetrics and Gynecology, West China Second University Hospital, Sichuan University, Chengdu, China

**Keywords:** levonorgestrel-releasing intrauterine system, LNG-IUS, heavy menstrual bleeding, systematic review, meta-analysis

## Abstract

**Introduction:**

To compare efficacy and safety of the levonorgestrel-releasing intrauterine system (LNG-IUS) with medical treatments for women with heavy menstrual bleeding.

**Materials and methods:**

We searched PubMed, Embase, the Cochrane Central Register of Controlled Trials, China National Knowledge Infrastructure (CNKI), and Wanfang databases for relevant randomized controlled trials (RCTs) in November 2021. All meta-analyses were performed using the random-effects model. PROSPERO registration number: CRD42021295379.

**Results:**

A total of trials (with 14 references) reporting on 1,677 women were included in this systematic review. The majority of the included RCTs were rated with low-to-unclear risk of bias in selection, detection, attrition, reporting, and other bias. All RCTs were rated as high risk in performance bias because blinding was difficult to ensure in the compared groups. Results of meta-analyses revealed that the number of clinical responders was greater in the LNG-IUS group than that in the medical treatments group at both 6-month (steroidal: five RCTs; *n* = 490; risk ratio [RR]: 1.72 [1.13, 2.62]; *I*^2^ = 92%; nonsteroidal: one RCT; *n* = 42; RR: 2.34 [1.31, 4.19]) and 12-month (steroidal: three RCTs; *n* = 261; RR: 1.31 [1.01, 1.71]; *I*^2^ = 74%) endpoints, with no clear differences on number of dropouts, and the incidence of adverse events.

**Conclusion:**

Evidence indicates that LNG-IUS is superior to the medical treatments in short-term and medium-term clinical responses, blood loss control, compliance, and satisfaction. Meanwhile, frequency of adverse events related to LNG-IUS is acceptable.

**Systematic review registration:**

PROSPERO, identifier CRD42021259335, https://www.crd.york.ac.uk/prospero/display_record.php?ID=CRD42021295379.

## Introduction

Heavy menstrual bleeding is a common condition in women of childbearing age, and ~30% of women are negatively affected during their reproductive years; it can cause serious impacts on physical health, emotional life, and quality of social life among women (1–4). However, based on subjective assessment or self-assessment, the incidence of heavy menstrual bleeding is higher at 24–52% in the United Kingdom, 4–27% in developing countries, 21% in Australia, and 18.2% in Beijing (4, 5). Conventional effective treatments for heavy menstrual bleeding include medical treatments and surgery. Medical treatments mainly include oral hormonal drugs (such as norethisterone and combined oral contraceptives) and tranexamic acid, which must be taken for a long-term period. Patients taking medical treatments are prone to poor compliance or missed doses, which impacts the treatment effect (6).

The levonorgestrel-releasing intrauterine system (LNG-IUS) is a highly effective sustained release system of intrauterine progesterone. It comprises 52 mg of levonorgestrel, which is released at a rate of approximately 20 μg/day during the first year. In addition to contraception, LNG-IUS is also approved for the treatment of disorders such as heavy menstrual bleeding (7). The mechanism of action of LNG-IUS for the treatment of heavy menstrual bleeding is significant inhibitory effect on the endometrium by a high concentration of progesterone in the uterine cavity. The levonorgestrel causes endometrial atrophy and makes endometrium insensitive to estrogens (8, 9), which can significantly reduce the amount of menstrual bleeding and the number of bleeding days (10). In the 2018 version of the guidelines for the diagnosis and treatment of heavy menstrual bleeding by the National Institute for Health and Clinical Excellence of the United Kingdom, LNG-IUS was recommended as the preferred drug for patients without obvious lesions, with fibroid diameters of <3 cm, without uterine cavity deformation, and with suspected or confirmed adenomyosis (8).

Existing systematic reviews on the treatment of heavy menstrual bleeding using LNG-IUS were either published several years ago (11–13) or focused on patients with idiopathic heavy menstrual bleeding (1). Therefore, this study aimed to conduct a comprehensive search of randomized controlled trials (RCTs) published to date and to systematically evaluate the efficacy and safety of LNG-IUS vs. medical treatments for primary or idiopathic heavy menstrual bleeding.

## Materials and methods

The protocol for this review has been registered in the International Prospective Register of Systematic Reviews (PROSPERO; registration number: CRD42021295379). The systematic review was reported in accordance with the standards described in the *Cochrane Handbook for Systematic Reviews of Interventions* and reported in line with the Preferred Reporting Items for Systematic Reviews and Meta-Analyses (PRISMA) standard (14).

### Eligibility criteria, information sources, and search strategy

We searched PubMed, Embase, the Cochrane Central Register of Controlled Trials, China National Knowledge Infrastructure (CNKI), and Wanfang databases from the establishment of the databases to November 2021, with no limitations on languages, regions, or publication years. The following terms were used and adapted for the searches in each database: (levonorgestrel releasing intrauterine system OR LNG-IUS OR mirena) AND (heavy menstrual bleeding OR menorrhagia). Detailed search strategies are presented in the Supplementary material 1.

We included relevant RCTs (in either English or Chinese) comparing LNG-IUS with medical treatments (monotherapy or multidrug combination, with no limitations on types, dosage regimens, or treatment durations) in women (aged ≥ 18 years) diagnosed with primary or idiopathic heavy menstrual bleeding. Primary outcome was clinical response to treatment (as defined in individual trials). Secondary outcomes included menstrual blood loss (MBL, measured using the pictorial blood loss assessment chart [PBAC], menorrhagia multiattribute scale [MMAS], or any other valid tools), and quality of life (measured using any validated scales such as health-related quality of life [HRQoL]-4), adverse events (such as headache, abdominal/pelvic pain, nausea, acne/hirsutism, back pain, spotting, genital discharge, dysmenorrhea, and depression), participant satisfaction, withdrawal of treatment, number of dropouts for any reason, amenorrhea, methemoglobin, and hemoglobin level. When possible, we divided all outcomes into short-term (≤6 months), medium-term (6–12 months), and long-term (>12 months) outcomes.

### Study selection

**Figure 1:**
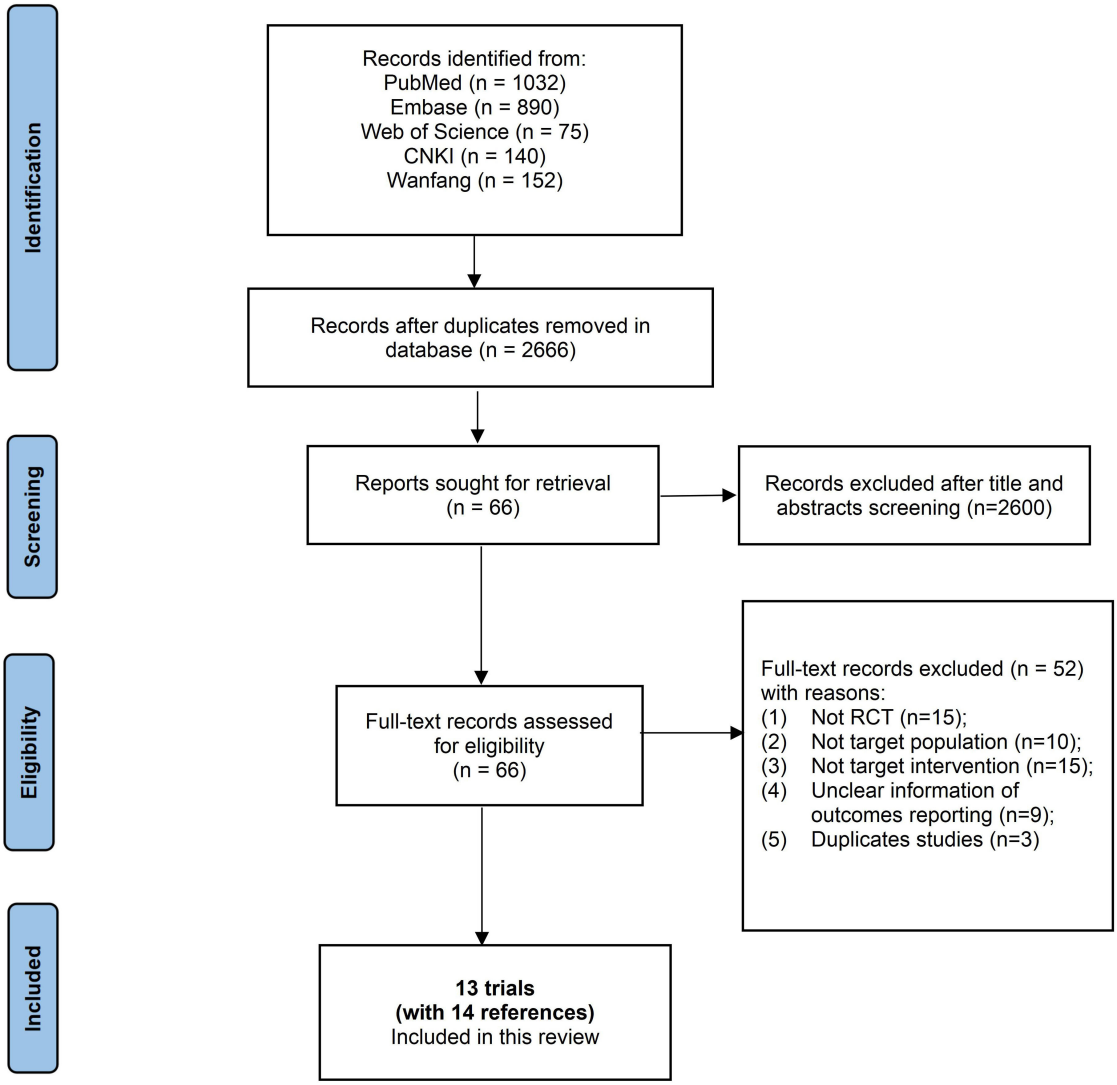
Study flow diagram.

Two reviewers (Chen SJ, Liu JH) independently inspected the search results to identify all potentially relevant references based on titles and abstracts, or full texts when necessary. Any disagreement during screening was resolved by discussion and, when necessary, with assistance from a third party. Finally, a PRISMA flow diagram is presented to illustrate the study selection process (Figure 1).

### Data extraction

Two reviewers (Liu JH, Peng SY) independently extracted data of study designs, characteristics of participants, description of interventions, and results of outcomes from all included RCTs using a standardized data extraction form (Supplementary Table 1).

### Assessment of risk of bias

**Figure 2:**
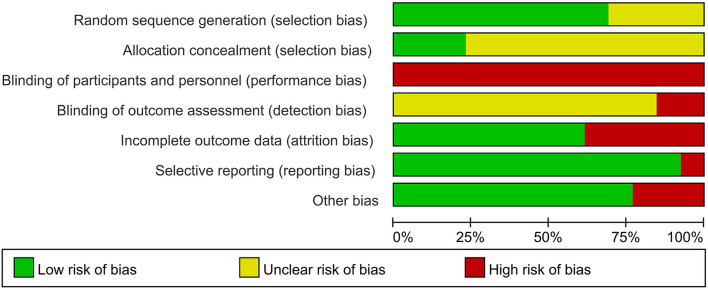
Risk of bias graph.

We assessed the risk of bias for included RCTs using the Cochrane Risk of Bias Tool, including sequence generation, allocation concealment, blinding of participants and personnel, blinding of outcome assessment, incomplete outcome data, and selective outcome reporting (15). We generated a risk of bias graph (Figure 2) and risk of bias summary (Supplementary Figure 1), wherein low risk was indicated in green, unclear risk in yellow, and high risk in red. Any disagreements were resolved by discussion with assistance from a third party when necessary.

### Data synthesis

For dichotomous outcomes, we estimated risk ratios (RRs) and their 95% confidence intervals (CIs). For continuous outcomes, we estimated mean differences (MDs) and their 95% CIs. We adopted the random-effects model for all meta-analyses using Review Manager 5.4.1 (16). We fully discussed clinical and methodological heterogeneity before meta-analysis, and we provided a descriptive summary of the outcome data when meta-analyses considering inappropriate. We defined *I*^2^ ≥ 50% accompanied by a statistically significant χ^2^ test (*p* < 0.1) as evidence of substantial levels of statistical heterogeneity. Except for different time points of outcome, we also performed meta-analysis separately based on whether steroids were used or not. Publication bias was also not investigated due to insufficient data well (no outcomes for which there were > 10 trials).

## Results

### Study selection

The database search resulted in the identification of 3,388 references, and no additional references were identified through other sources. A total of 2,666 unique records remained after de-duplication. Among these, we excluded 2,600 unique records upon inspection of titles and abstracts. We read the remaining 66 unique records completely and subsequently excluded 52 unique records with reasons (details in Figure 1). Finally, 13 trials [with the remaining 14 references (17–30)] were included in this systematic review, and 12 trials were included in meta-analyses (Figure 1).

### Study characteristics

A total of 13 trials (with 14 references) involving 1,677 women were included [Dong (17), Endrikat et al. (18), Gupta et al. (19, 20), Irvine et al. (21), Kaunitz et al. (22), Kavasoglu et al. (23), Kiseli et al. (24), Liu (25), Malik et al. (26), Reid and Susanna (27), Shabaan et al. (28), Zhao and Hongyan (29), and Zhong et al. (30)]. Overall, 10 single-center trials were included, of which four were conducted in China (17, 25, 29, 30) and the remaining six were conducted in Scotland (21), Turkey (24), the United Kingdom (27), Pakistan (26), and Egypt (28). The remaining three multicenter trials (18, 20, 22) recruited participants from Canada, the United Kingdom, the United States, and Brazil. Sample sizes ranged from 42 (18) to 571 (20). Women diagnosed with heavy menstrual bleeding were all included [among which was idiopathic heavy menstrual bleeding (17, 18, 21, 23, 26–29)]. The average age of the women ranged from 28.3 to 41.9 years. As reported, the average body mass index ranged from 21.8 to 29.2 kg/m^2^, and the median PBAC score at baseline ranged from 228 to 300. In terms of participants' baseline characteristics, most trials did not report information on menstrual cycle length, menstrual period, or number of births. Kaunitz et al. (22) reported that the mean menstrual cycle length was 2.6 days and the mean number of births was 2.5. Endrikat et al. (18) reported that six participants had no children, 10 had one child, 16 had two children, and seven had more than three children (details in Table 1).

**Table 1:** Characteristics of included trials.

**References**	**Location**	**Center**	**Diagnosis**	**Medical treatment used in control group**	**Sample size at randomization, n**		**Mean age (year)**	**Mean BMI (kg/m2)**	**Mean PBAC score**	**Follow-up (months)**	**Outcomes**
					**LNG-IUS**	**Medical treatment**					
Dong (17)	China	1	Heavy Menstrual Bleeding	norethisterone	40	40	28.86	21.82	NR	1; 6	1) Clinical response 2) Menstrual blood loss 3) Withdrawal of treatment 4) Number of drop-out
Endrikat et al. (18)	Canada	9	Idiopathic Menorrhagia	contraceptive pill (norethindrone acetate+ethinyl estradiol)	22	20	42.1	23.5	median LNG-IUS: 228 Control: 290	3; 6; 9; 12	1) Clinical response to treatment (Treatment success) 2) Menstrual blood loss (PBAC) 3) Quality of life (menorrhagia severity score) 4) Hemoglobin 5) Withdrawal of treatment 6) Number of drop-out 7) Adverse events
Gupta et al. (20)	England	63	Heavy Menstrual Bleeding	mefenamic acid, tranexamic acid, norethisterone, a combined estrogen–progestogen or progesterone-only oral contraceptive pill (any formulation), or medroxyprogesterone acetate injection	285	286	41.9	29.2	NR	6; 12; 24; 60	1) Menstrual blood loss (MMAS) 2) Quality of life (SF-36/EQ-5D/SAQ) 3) Withdrawal of treatment 4) Number of drop-out
Irvine et al. (21)	Scotland	1	Idiopathic Menorrhagia	norethisterone	22	22	median (range) LNG-IUS: 38.5 (31–45) Control: 39 (30–45)	NR	NR	1; 3	1) Menstrual blood loss (alkaline hematin method) 2) Patients satisfaction 3) Amenorrhea 4) Hemoglobin 5) Withdrawal of treatment 6) Number of drop-out 7) Adverse events
Kaunitz et al. (22)	United States, Canada, and Brazil	55	Heavy Menstrual Bleeding	oral medroxyprogesterone acetate	82	83	38.8	27.3	NR	3; 6	1) Clinical response to treatment (Treatment success) 2) Menstrual blood loss (alkaline hematin method) 3) Number of drop-out 4) Adverse events
Kavasoglu and Ahmet (23)	Turkey	1	Heavy Menstrual Bleeding	norethisterone acetate	97	95	40.3	NR	NR	6; 12	1) Menstrual blood loss (VBS) 2) Hemoglobin 3) Withdrawal of treatment 4) Number of drop-out 5) Adverse events
Kiseli et al. (24)	Turkey	1	Heavy Menstrual Bleeding	norethisterone; tranexamic acid	28	28; 28	42.1	NR	median (IQR) LNG-IUS: 300 (91.75) Control 1: 290 (87.50) Control 2: 300 (174)	1; 3; 6	1) Clinical response to treatment (PBAC scores <100) 2) Menstrual blood loss (PBAC) 3) Quality of life (WHOQOL-BREF TR) 4) Hemoglobin 5) Withdrawal of treatment 6) Number of drop-out 7) Adverse events
Liu (25)	China	1	Heavy Menstrual Bleeding	norethisterone	50	50	28.3	21.83	NR	6	1) Clinical response to treatment (total effective rate) 2) Menstrual blood loss 3) Withdrawal of treatment 4) Number of drop-out
Malik et al. (26)	Pakistan	1	Idiopathic Menorrhagia	norethisterone tablet	38	38	40.2	NR	NR	3; 6	1) Hemoglobin 2) Patient's acceptability 3) Withdrawal of treatment 4) Number of drop-out
Reid et al. (27)	England	1	Idiopathic Menorrhagia	Mefenamic acid	25	26	39	NR	median (range) LNG-IUS: 240 (91–545) Control: 233 (77–469)	3; 6	1) Menstrual blood loss (alkaline hematin method/PBAC) 2) Withdrawal of treatment 3) Number of drop-out 4) Adverse events
Shabaan et al. (28)	Egypt	1	Idiopathic Menorrhagia	contraceptive (ethinyl estradiol+levonorgestrel)	56	56	39	30.4	315.2	6; 12	1) Clinical response to treatment (treatment failure) 2) Menstrual blood loss (alkaline hematin method/PBAC) 3) Quality of life (HRQoL-4) 4) Amenorrhoea 5) Hemoglobin 6) Number of drop-out 7) Adverse events
Zhao et al. (29)	China	1	Heavy Menstrual Bleeding	desogestrel and ethlinylestraliol tablets + ibuprofen tablets + etamsylate tablets	25	25	30.32	NR	97.3	3	1) Menstrual blood loss (PBAC) 2) Hemoglobin 3) Amenorrhea 4) Withdrawal of treatment 5) Number of drop-out 6) Adverse events
Zhong et al. (30)	China	1	Heavy Menstrual Bleeding	desogestrel and ethlinylestraliol tablets	55	55	35.21	NR	NR	6; 12	1) Clinical response to treatment (total effective rate) 2) Menstrual blood loss 3) Withdrawal of treatment 4) Number of drop-out

### Risk of bias of included studies

A total of nine (17, 20–25, 27, 28) of the 13 trials provided a description of adequate random sequence generation (computer-generated lists of random numbers, central randomization system, or sortition), and three (21, 27, 28) of the 13 trials reported adequate concealment of allocation and were rated as low risk of selection bias. All trials were rated as high risk in performance bias because blinding was difficult to ensure in the compared groups. Two trials (27, 28) reported that the assessment of outcomes was nonblinded and were rated as high risk of detection bias. One trial (24) had a substantial rate of participant withdrawal (>20%) and was considered as high risk of attrition bias. The other trials were assessed as low risk of attrition bias because of no missing data, or low and balanced dropout rates and reasons for dropout across compared groups. All trials appropriately reported all the outcomes stated in their methods sections and were assessed as low risk of selective reporting bias. Three trials (18, 22, 27) were funded by industries and were therefore considered as having an unclear risk of bias (Figure 2 and Supplementary Figure 1).

### Synthesis of results

#### Clinical response

**Figure 3:**
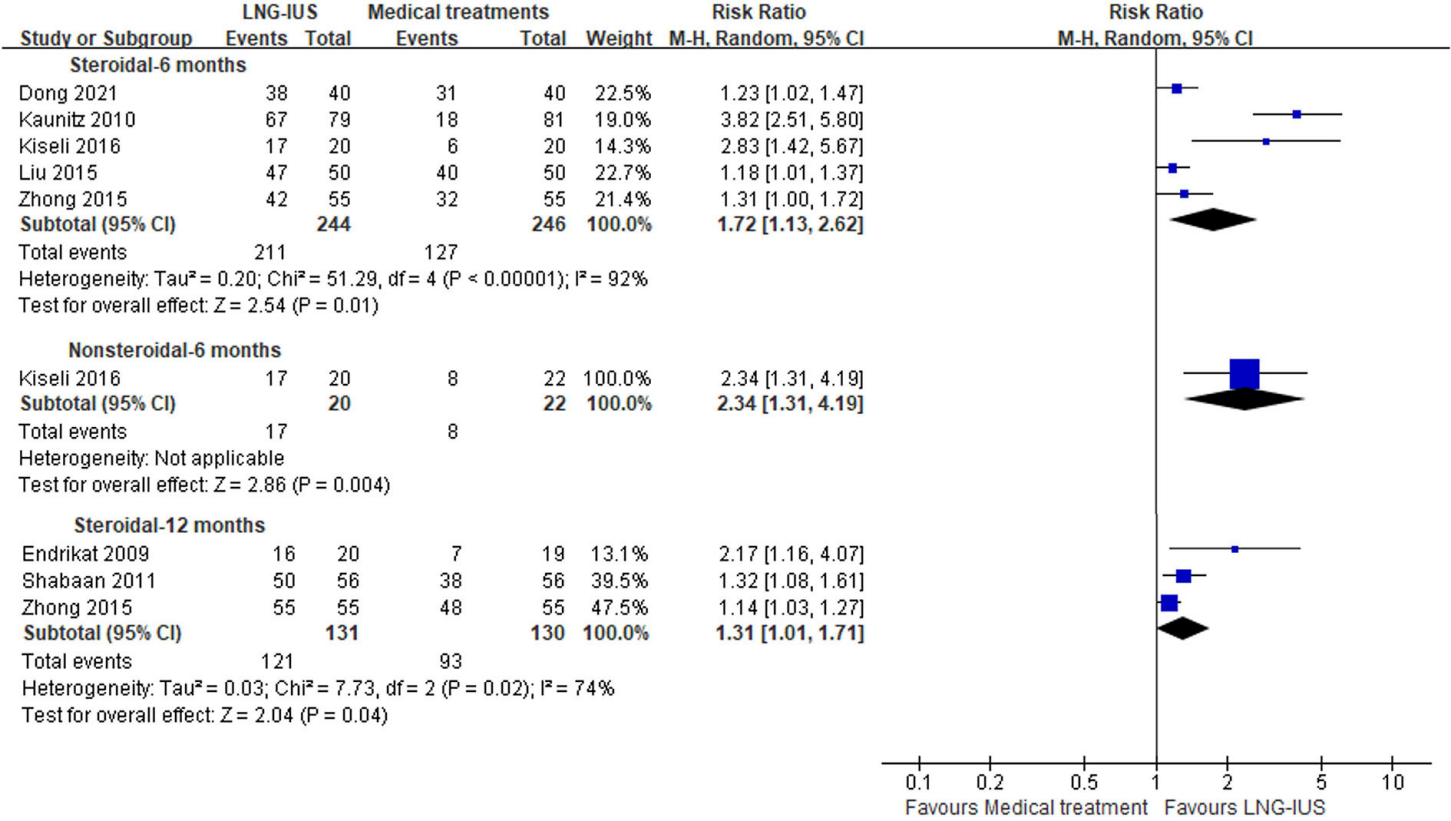
Forest plots of LNG-IUS compared with medical treatment for clinical response at 6 and 12 months.

Seven trials (17, 18, 22, 24, 25, 28, 30) reported clinical response and were included in this meta-analysis (Figure 3). Table 2 presents the definitions of clinical response in each trial. The results showed that the number of clinical responders was greater in the LNG-IUS group than that in the medical treatments group at 6 months, regardless of steroidal medical treatments or not [steroidal: five RCTs (17, 22, 24, 25, 30); *n* = 490; RR = 1.72; 95% CI = 1.13–2.62; *p* = 0.01; *I*^2^ = 92%; nonsteroidal: one RCT (24); *n* = 42; RR = 2.34; 95% CI = 1.31–4.19; *p* = 0.004; Figure 3]. At 12 months, the results showed that the number of clinical responders in the LNG-IUS group was more than that in steroidal medical treatments group [RCTs (18, 28, 30); *n* = 261; RR = 1.31; 95% CI = 1.01–1.71; *p* = 0.04; *I*^2^ = 74%; Figure 3]. No obvious source of the substantial heterogeneity was identified. We still performed this meta-analysis because of the consistency of all included RCTs.

**Table 2:** Definition of clinical response.

**Study**	**Medical treatments**	**Definition**
Dong (17)	Norethisterone	Significant improvement: After treatment, menstrual volume was normal and symptoms disappeared. Improvement: After treatment, menstrual volume decreased and symptoms improved, with a little bleeding or amenorrhea. No improvement: Above results were not achieved after treatment. Total efficacy = significant improvement + improvement
Endrikat et al. (18)	Norethindrone acetate and ethinyl estradiol	Treatment success (i.e., clinical outcome) was defined as MBL of <100 mL at 12 months, and treatment failure was defined as MBL of 100 mL or if the treatment was discontinued.
Kaunitz et al. (22)	Medroxyprogesterone acetate	Treatment success was defined as MBL of <80 mL at the end of the study and reduction of ≥50% in MBL from baseline.
Kiseli et al. (24)	Two groups: - Norethisterone - Tranexamic acid	PBAC scores of <100
Liu et al. (25)	Norethisterone	Significant improvement was defined as MBL returned to normal and disappearance of clinical symptoms. Improvement was defined as amenorrhea or spotting bleeding, menstrual volume decreased, obvious improvement on clinical symptoms. No clinical response was defined as no significant decrease or increase in MBL and no improvement in clinical symptoms compared with that before treatment. Total efficacy = significant improvement + improvement
Shabaan et al. (28)	Levonorgestrel and ethinyl estradiol	Treatment failure was defined as the initiation of an alternative medical treatment or the need for surgery.
Zhong et al. (30)	Desogestrel and ethinyl estradiol	Cure was defined as decreased MBL, resumed regular menstrual cycle, no obvious abdominal pain or severe discomfort, and hemoglobin levels returned to normal. Improvement was defined as decreased MBL, menstrual cycle returned to normal but with prolonged period, and no obvious discomfort. No clinical response was defined as nonsignificant improvement in MBL, unstable menstrual cycle, prolonged menstrual cycle, and nonsignificant improvement in hemoglobin levels. Total efficacy = cure + improvement

#### Menstrual blood loss

Meta-analysis was not performed because the included RCTs used different measurement tools. MBL was measured using PBAC in four trials (18, 24, 27, 28) (Table 3 and Supplementary Table 2). Kiseli et al. (24) (*n* = 62, vs. NETA and vs. tranexamic acid) and Shabaan et al. (28) (*n* = 112, vs. levonorgestrel combined with ethinyl estradiol) reported that the percentage reduction in PBAC scores was greater in the LNG-IUS group than that in the medical treatment groups at 6 months. Reid and Susanna (27) (*n* = 51, vs. tranexamic acid) reported that the PBAC scores at endpoint were lower in the LNG-IUS group than that in the medical treatment group at 6 months. Endrikat et al. (18) (*n* = 39, vs. norethindrone acetate combined with ethinyl estradiol) and Shabaan et al. (28) (*n* = 112, vs. levonorgestrel combined with ethinyl estradiol) also reported that the percentage reduction in PBAC scores was greater in the LNG-IUS group than that in the medical treatments group at 12 months. MBL was measured using visual blood score (VBS) in one trial [Kavasoglu et al. (23) (*n* = 192, vs. norethisterone acetate)], which reported that the VBS scores at endpoint were lower in the LNG-IUS group than that in the medical treatment group at 6 and 12 months. MBL was measured using the alkaline hematin method in four trials (21, 22, 27, 28) (Table 3 and Supplementary Table 3), and similar results were identified in that the MBL was lower in the LNG-IUS group than in the medical treatments group at 3, 6, or 12 months. Gupta 2015 (20) (*n* = 571; vs. usual medical treatment) measured MBL using MMAS at 2 and 5 years and reported that the average MMAS score was greater in the LNG-IUS group than in the medical treatments group (MD = 13.4; 95% CI = 9.9–16.9; *p* < 0.001) at 2 years; however, no clear difference was identified between the compared groups at 5 years (MD = 3.9; 95% CI = −0.6 to 8.3; *p* = 0.09). Data of other measurement methods and time points of measurement are presented in Supplementary Table 3.

**Table 3:** Results summary on menstrual blood loss (MBL).

**Follow-up**	**Results**	**Study**	**Total number in analysis**	**LNG-IUS**	**Medical treatment**	***P*-Value**
**MBL assessed by pictorial bleeding assessment chart scores**						
6-month	Reduction in PBAC score, %, median	Kiseli et al. (24)	LNG-IUS: n=20 NETA: n=20 Tranexamic acid: n=22	85.8	NETA: 53.1 tranexamic acid: 60.8	P<0.001
	Reduction in PBAC score, %, mean±SD	Shabaan et al. (28)	LNG-IUS: n=56 Levonorgestrel combined with ethinyl estradiol: n=56	89.5 ± 11.7	41.6 ± 53.6	p<0.001
	PBAC score, median (range)	Reid and Susanna (27)	LNG-IUS: n=25 Mefenamic acid: n=26	25 (0–402)	159 (50–307)	p<0.001
12-month	Reduction in PBAC score, %, median	Endrikat et al. (18)	LNG-IUS: n=20 Norethindrone acetate combined with ethinyl estradiol: n=19	83	68	p=0.002
	Reduction in PBAC score, %, mean±SD	Shabaan et al. (28)	LNG-IUS: n=56 Levonorgestrel combined with ethinyl estradiol: n=56	86.6 ± 17.0	2.5 ± 93.2	p<0.001
**MBL assessed by alkaline haematin method**						
3-month	MBL, ml, median (range)	Irvine et al. (21)	LNG-IUS: n=22 Norethisterone: n=22	6 (0-284)	20 (4–137)	P=0.03
6-month	Reduction in MBL, %, mean (SD)	Kaunitz et al. (22)	LNG-IUS: n=82 Medroxyprogesterone acetate: n=83	70.8 ± 88.3	21.5 ± 35.8	P<0.001
	MBL, ml, median (range) MBL,	Reid and Susanna (27)	LNG-IUS: n=25 Mefenamic acid: n=26	5 (0–45)	100 (46–168)	P<0.001
	ml, mean±SD	Shabaan et al. (28)	LNG-IUS: n=56 Levonorgestrel combined with ethinyl estradiol: n=56	44.4 ± 34.9	118.2 ± 75.0	P<0.001

#### Health-related quality of life

Only four trials (18, 20, 24, 28) reported on HRQoL using various scales (details in Supplementary Table 4); therefore, meta-analysis was inappropriate.

Endrikat et al. (18) reported on HRQoL using menorrhagia severity scores (ranging from 0 to 100%, wherein high scores indicated poorer outcomes). The results showed that the menorrhagia severity scores at 6 months were lower in the LNG-IUS group than that in the medical treatment (norethindrone acetate combined with ethinyl estradiol) group (MD = −6.37; 95% CI = −12.61 to −0.14); however, no clear difference was identified at other time points (i.e., 3, 9, or 12 months).

Gupta et al. (20) reported on HRQoL using the following scales: the Short Form 36 Health Survey Questionnaire (SF-36; scores ranging from 0 to 100, wherein high scores indicated good outcomes), the European Quality of Life-5 Dimensions (EQ-5D; scores ranging from −0.59 to 100, wherein high scores indicated good outcomes), the EQ-5D visual analog scale (scores ranging from 0 to 100, wherein high scores indicated good outcomes), and the Sexual Activity Questionnaire (pleasure subscale scores ranging from 0 to 18, wherein high scores indicated good outcomes; discomfort subscale scores ranging from 0 to 6, wherein high scores indicated poor outcomes; and habit assessed relative to perceived usual activity as an ordinal response). The results showed that the SF-36 subscale scores (including physical role, social functioning, energy/vitality, and pain) at 6 months, 12 months, or 2 years were higher in the LNG-IUS group than that in the usual medical treatment group. No clear difference was identified in other subscales assessed using other tools. Detailed data are presented in Supplementary Table 5.

Kiseli et al. (24) reported on HRQoL using the World Health Organization Quality of Life, Short Form, Turkish version (WHOQOL-BREF TR; scores ranging from 1 to 100, wherein high scores indicated good outcomes). The results indicated that the HRQoL in physical aspects increased in the LNS-IUS group and tranexamic acid group, whereas there was no clear difference in norethisterone acid, when pre- and post-treatment scores were compared. However, there was no clear difference in the changes in WHOQL-BREF TR scores at 6 months from baseline between the LNG-IUS and medical treatment groups (tranexamic acid or norethisterone acid), including the physical, psychological, social, and environmental domains. Detailed data are shown in Supplementary Table 6. Shabaan et al. (28) reported on HRQoL using HRQoL-4 and showed that the QoL of women improved in both groups after treatment (including the reduction in the physically days but not the unhealthy days related to mental health in the both groups; detailed data are shown in Supplementary Table 7). This study also reported that a significant reduction in the number of lost days in the LNG-IUS group at 6 and 12 months.

### Other outcomes

Table 4 summarizes the results of other secondary outcomes except for methemoglobin level, which was not reported in included studies.

**Table 4:** Results summary of meta-analyses for secondary outcomes.

**Outcomes**	**RCTs (n)**	**Participants (n)**	**RR [95% CI]**	**I^2^**
**Adverse events**				
Steroidal-6 months	1	42	1.30 [0.61, 2.76]	NA
Nonsteroidal-6 months	1	44	1.00 [0.52, 1.91]	NA
Steroidal-12 months	1	39	0.95 [0.75, 1.21]	NA
**Serious adverse events**				
Nonsteroidal-6 months	1	51	5.19 [0.26, 103.07]	NA
Mixed-60 months	1	571	0.91 [0.63, 1.30]	NA
**Withdrawal of treatment**				
Steroidal-3 or 6 months	4	863	**0.43 [0.31, 0.60]**	0%
Nonsteroidal-6 months	2	107	1.18 [0.43, 3.29]	0%
Steroidal-12 months	2	234	**0.47 [0.29, 0.78]**	0%
Mixed-12 months	1	571	**0.48 [0.35, 0.65]**	NA
Mixed-24 months	1	571	**0.49 [0.39, 0.60]**	NA
Mixed-60 months	1	571	**0.56 [0.48, 0.65]**	NA
**Number of drop-out**				
Steroidal-3 or 6 months	4	457	0.58 [0.33, 1.03]	42%
Nonsteroidal-6 months	2	107	1.12 [0.54, 2.32]	0%
Mixed-6 months	1	571	0.67 [0.40, 1.12]	NA
Steroidal-12 months	3	346	**0.58 [0.38, 0.88]**	0%
Mixed-12 months	1	571	0.73 [0.47, 1.14]	NA
Mixed-24 months	1	571	0.69 [0.47, 1.01]	NA
Mixed-60 months	1	571	0.89 [0.67, 1.17]	NA
**Satisfaction of participants**				
Steroidal-3 or 6 months	3	158	1.11 [0.92, 1.35]	0%
Nonsteroidal-6 months	1	44	1.21 [0.82, 1.79]	NA
Steroidal-12 months	1	37	1.38 [0.91, 2.09]	NA
**Amenorrhoea**				
Steroidal-3 or 6 months	2	81	1.12 [0.02, 61.74]	75%
Steroidal-12 months	1	95	14.69 [0.86, 250.22]	NA

RCT, randomized controlled trial; RR, risk ratio; “Mixed” stands for comparator group, including both steroidal medical treatment and nonsteroidal medical treatment (20).

Seven trials reported the total number of overall (18, 21–24, 27, 29) or serious adverse events (20, 27) (Table 4), and no clear difference was identified between the compared groups at 6 and 12 months, regardless of steroidal medical treatments or not. The common specific adverse events reported by the included trials were abdominal pain, breast tenderness, headache, intermenstrual bleeding, nausea, ovarian cyst, and increased weight, and no clear differences were identified (Supplementary Figures 2–8).

Six trials (18, 20, 21, 23, 24, 27) reported the number of participants who withdrew from treatment (i.e., discontinued the treatment), and the data showed that fewer participants withdrew from treatment in the LNG-IUS group than in the steroidal medical treatment group at the short-term (3 or 6 months) and medium-term (12 months) follow-ups. Three trials (18, 20–24, 27, 28) reported number of dropouts, and found less dropouts in the LNG-IUS group than in steroidal medical treatment group at 12 months. Four trials (18, 21, 24, 26) reported participants satisfaction and found that more participants satisfied in the LNG-IUS group than that in the medical treatment groups, although no significant statistical difference was identified due to very small sample size. Amenorrhea was identified in both the LNG-IUS group and steroidal medical treatment group, and no clear differences were found. Five trials (18, 23, 26, 28, 29) reported hemoglobin levels and obtained different results based on different comparisons and follow-up durations (Supplementary Table 8). Endrikat et al. (18) (n = 39, vs. norethindrone acetate combined with ethinyl estradiol) found no clear difference in the change in hemoglobin levels from baseline to 12 months between the compared groups. Zhao et al. (29) (n = 50, vs. desogestrel combined with ethinyl estradiol) reported that the level of hemoglobin in the LNG-IUS group was significantly higher than that in the steroidal medical treatment group at the end of 3 months. Kavasoglu et al. (23) (n = 192, vs. norethisterone) and Malik et al. (26) (n = 76, vs. norethisterone) reported that the level of hemoglobin in the LNG-IUS group was higher than that in the norethisterone group at the end of 6 months. Kavasoglu et al. (23) (n = 192, vs. norethisterone) and Shabaan et al. (28) (n = 112, vs. levonorgestrel combined with ethinyl estradiol) reported that women in the LNG-IUS group had higher hemoglobin levels at the end of 12 months than those in the steroidal medical treatment group.

## Discussion

### Main findings

This systematic review summarized the results of 13 RCTs that involved 1,677 women (average age ranging from 27.8 to 43.2 years) with heavy menstrual bleeding and investigated the efficacy and safety of LNG-IUS. The findings of this review showed that the number of clinical responders was greater in the LNG-IUS group than that in the medical treatment group at both the 6- and 12-month endpoints. Data showed that LNG-IUS reduces participants' MBL at both the 6- and 12-month endpoints. LNG-IUS has a positive effect on HRQoL (such as physical role, social functioning, energy/vitality, and pain), although high-quality evidence was needed to draw a firm conclusion. In terms of treatment withdrawal, the data showed that fewer participants discontinued treatment in the LNG-IUS group than that in the steroidal medical treatment group at the short-term and medium-term follow-ups. This suggests that compliance with treatment was better in the LNG-IUS group than that in the steroidal medical treatment group and that missed doses of medical treatment is likely to occur in the process of treatment. Current evidence also showed that the percentage of satisfaction was higher in the LNG-IUS group than that in medical treatment group, although no significant statistical difference was identified between two compared groups. Amenorrhea only occurred in the LUG-IUS group and no significant statistical difference was identified as well due to very small sample size. There were no clear differences between the groups for the number of dropouts and most adverse events.

Compared to the previous reviews (1), this systematic review updated available current evidence and focused on patients with heavy menstrual bleeding (not only included patients with idiopathic heavy menstrual bleeding). There is another systematic review assessing the effectiveness, acceptability, and safety of progestogen-releasing intrauterine devices in reducing heavy menstrual bleeding (31). Rodriguez et al. found that the LNG-IUS may improve heavy menstrual bleeding and HRQoL, and has similar serious adverse events when compared to other medical therapy. In Rodriguez et al. (32), by systematically assessing and summarizing the evidence from studies included in Cochrane Reviews on treatment for heavy menstrual bleeding, LNG-IUS was suggested as the best first-line treatment for reducing MBL. In addition, Oderkerk et al. (33) suggested that inserting an LNG-IUS immediately after endometrial ablation/resection seems to lower the hysterectomy and reintervention rates compared with ablation/resection alone among patients, which also demonstrated the importance of LNG-IUS in the therapy of heavy menstrual bleeding. Overall, evidence showed that the conclusions of the present systematic review are similar to those reported by previous reviews.

### Strengths and limitations

This systematic review strictly followed the standards of the *Cochrane Handbook for Systematic Reviews of Interventions*. The protocol was registered, and the search was comprehensive, thereby minimizing the bias in the production process. The overall risk of bias for the included studies in this systematic review was moderate. All the included trials had a high risk of performance bias due to the blinding of participants and personnel, which is likely to be difficult to ensure when comparing LNG-IUS with medical treatment. Objective outcomes should be considered in further research (e.g., not using self-reported scales). In addition, the meta-analyses of some outcomes were not applicable because the included studies used varying definitions or measurement tools for the same outcome. For example, there is some confusion about the definition and scope of “abnormal” uterine bleeding in the field of gynecology (34). Similar terms are used in different ways in different countries or even by different gynecologists in a single clinical setting, which may affect the application of relevant evidence in clinical practice. Therefore, more rigorous, well-designed RCTs or observational studies are needed to draw certain conclusions.

## Conclusion

Evidence indicates that LNG-IUS is superior to medical treatment for women with heavy menstrual bleeding. The LNG-IUS is a more effective therapy, with low incidence of adverse events and high patient compliance, which increases the ease of use in clinical practice. The available studies have some limitations in terms of study design and the manner in which information on patient outcomes was reported, indicating that the evidence continues to be limited to a certain extent.

## Data availability statement

The original contributions presented in the study are included in the article/Supplementary material, further inquiries can be directed to the corresponding author.

## Author contributions

SC and YZ contributed to review—writing and revising. All authors reviewed and approved the final manuscript and contributed to protocol development, study selection, and data collection.

## Conflict of interest

The authors declare that the research was conducted in the absence of any commercial or financial relationships that could be construed as a potential conflict of interest.

## Publisher's note

All claims expressed in this article are solely those of the authors and do not necessarily represent those of their affiliated organizations, or those of the publisher, the editors and the reviewers. Any product that may be evaluated in this article, or claim that may be made by its manufacturer, is not guaranteed or endorsed by the publisher.
