# Supplementary material for: LNG-IUS vs. medical treatments for women with heavy menstrual bleeding: A systematic review and meta-analysis
Source: Front Med (Lausanne). 2022 Aug 25;9:948709. doi: 10.3389/fmed.2022.948709 (PMC9452891; doi:10.3389/fmed.2022.948709)
Supplement: Supplementary file 1 [file Data_Sheet_1.docx]

**Methods S1. Search strategies**

**Table S1. Data extraction form**

**Table S2. Summary results of pictorial bleeding assessment chart (PBAC) scores**

**Table S3. Summary results of menstrual blood loss (MBL) parameters**

**Table S4. Detailed description on scales using to assess health-related quality of life (HRQoL)**

**Table S5. Summary results of HRQoL assessed by the short form-questionnaire-36 (SF-36) version 2, the European Quality of Life-5 Dimensions (EQ-5D) and the EQ-5D VAS, the Sexual activity questionnaire (SAQ)**

### Table S6. Summary results of HRQoL assessed by WHOQOL-BREF TR

### Table S7. Summary results of HRQoL assessed by f HRQoL-4

### Table S8. Summary results of hemoglobin

**Figure S1. ‘Risk of bias’ summary**

**Figure S2. Forest plots of meta-analysis of LNG-IUS compared with medical treatments for abdominal pain**

**Figure S3. Forest plots of meta-analysis of LNG-IUS compared with medical treatments for breast tenderness**

**Figure S4. Forest plots of meta-analysis of LNG-IUS compared with medical treatments for headache**

**Figure S5. Forest plots of meta-analysis of LNG-IUS compared with medical treatments for intermenstrual bleeding**

**Figure S6. Forest plots of meta-analysis of LNG-IUS compared with medical treatments for nausea**

**Figure S7. Forest plots of meta-analysis of LNG-IUS compared with medical treatments for ovarian cyst**

**Figure S8. Forest plots of meta-analysis of LNG-IUS compared with medical treatments for weight increase**

**Methods S1. Search strategies**

A. Pubmed

1. mirena

2. levonorgestrel releasing intrauterine system

3. LNG-IUS

4. levonorgestrel intrauterine system

5. 1 or 2 or 3 or 4

6. menorrhagia

7. menorrhea

8. heavy menstrual bleeding

9. 6 or 7 or 8

10. 5 and 9

B. EMBASE and CENTRAL

1. mirena.mp. or mirena/

2. levonorgestrel releasing intrauterine system/ or levonorgestrel releasing intrauterine system.mp.

3. LNG-IUS.mp. or LNG-IUS/

4. levonorgestrel intrauterine system.mp. or levonorgestrel intrauterine system/

5. 1 or 2 or 3 or 4

6. menorrhagia.mp. or menorrhagia/

7. menorrhea.mp. or menorrhea/

8. heavy menstrual bleeding.mp. or heavy menstrual bleeding/

9. 6 or 7 or 8

10. 5 and 9

C. CNKI

(SU='曼月乐'+'左炔诺孕酮宫内节育系统'+'左炔诺孕酮宫内缓释系统') AND (SU='月经过多')

D. Wanfang Data

主题:( 曼月乐+左炔诺孕酮宫内节育系统+左炔诺孕酮宫内缓释系统) and 主题:(月经过多)

**Table S1. Data extraction form**

| **Study ID** | |
| --- | --- |
| **Contact Info** | |
| **Methods** | Country/Location |
|  | Centre (Single/Multi/NR] |
| **Participants** | Diagnosis |
|  | Sample size at randomization |
|  | Mean age (years) |
|  | Mean BMI/(kg/m2) |
|  | Menstrual cycle (days) |
|  | Menstrual period (days) |
|  | Burst size |
|  | Pictorial blood loss assessment chart score |
|  | Inclusion criteria |
|  | Exclusion criteria |
| **Interventions** | Sample size of LNG-IUS at randomization |
|  | Description of LNG-IUS |
|  | Dosage/frequency/Duration of LNG-IUS |
|  | Sample size of medical treatments at randomization |
|  | Description of medical treatments |
|  | Dosage/frequency/Duration of medical treatments |
| **Outcomes** | Outcome name |
|  | Definitions/Measurement of outcome |
|  | Timepoint of assessment |
|  | Outcomes (unable to use/not predefined in protocol) |
| **Outcome data** | |
| **Dichotomous data** (such as AE) | Events number in LNG-IUS group |
|  | Total number in LNG-IUS group |
|  | Events number in medical treatments group |
|  | Total number in medical treatments group |
| **Continuous data** (such as QoL) | Mean of LNG-IUS group |
|  | SD of LNG-IUS group |
|  | Total number in LNG-IUS group |
|  | Mean of medical treatments group |
|  | SD of medical treatments group |
|  | Total number in medical treatments group |
| **ROB assessment of included studies** | |
| **Randomization** | Support for judgement |
| **Allocation concealment** | Support for judgement |
| **Blinding of participants and personnel** | Support for judgement |
| **Blinding of outcome assessor** | Support for judgement |
| **Incomplete data** | Support for judgement (drop-out) |
| **Selective reporting** | Support for judgement |
| **Other bias** | Support for judgement (study funding) |

**Table S2. Summary results of pictorial bleeding assessment chart (PBAC) scores**

| **Follow-up** | **Results** | **study** | **Sample Size** | **LNG-IUS** | **Medical treatments** | **Conclusion/comment** |
| --- | --- | --- | --- | --- | --- | --- |
| at 3-month | Reduction in PBAC score, %, median | Kiseli 2016 | LNG-IUS: n=20 NETA: n=20 Tranexamic acid: n=22 | 66.2 | NETA: 47.4 tranexamic acid: 49.3 | p＞0.05 |
|  | PBLAC score, median (range) | Reid 2005 | LNG-IUS: n=25 Mefenamic acid: n=26 | 49 (0–286) | 161 (77–262) | p＜0.001 |
| at 6-month | Reduction in PBAC score, %, median | Kiseli 2016 | LNG-IUS: n=20 NETA: n=20 Tranexamic acid: n=22 | 85.8 | NETA: 53.1 tranexamic acid: 60.8 | p＜0.001 |
|  | Reduction in PBAC score, %, mean±SD | Shabaan 2011 | LNG-IUS: n=56 Levonorgestrel combined with ethinyl estradiol: n=56 | 89.5±11.7 | 41.6±53.6 | p＜0.001 |
|  | PBLAC score, median (range) | Reid 2005 | LNG-IUS: n=25 Mefenamic acid: n=26 | 25 (0–402) | 159 (50–307) | p＜0.001 |
| at 12-month | Reduction in PBAC score, %, median | Endrikat 2009 | LNG-IUS: n=20 Norethindrone acetate combined with ethinyl estradiol: n=19 | 83 | 68 | p= 0.002 |
|  | Reduction in PBAC score, %, mean±SD | Shabaan 2011 | LNG-IUS: n=56 Levonorgestrel combined with ethinyl estradiol: n=56 | 86.6±17.0 | 2.5±93.2 | p＜0.001 |

**Table S3. Summary results of menstrual blood loss (MBL) parameters**

| **Follow-up** | **Results** | **study** | **Sample Size** | **LNG-IUS** | **Medical treatment** | **Conclusion/comment** |
| --- | --- | --- | --- | --- | --- | --- |
| at 1-month | MBL, ml, median (range) | Irvine 1998 | LNG-IUS: n=22 Norethisterone: n=22 | 16 (0-62) | 46 (0-213) | P=0.02 |
|  | MBL, ml, mean±SD | Dong 2021 | LNG-IUS: n=40 Norethisterone: n=40 | 34.30±7.45 | 50.82±8.75 | P＜0.05 |
| at 3-month | MBL, ml, median (range) | Irvine 1998 | LNG-IUS: n=22 Norethisterone: n=22 | 6 (0-284) | 20 (4-137) | P=0.03 |
|  |  | Reid 2005 | LNG-IUS: n=25 Mefenamic acid: n=26 | 12 (0–240) | 94 (29–219) | P＜0.001 |
|  | MBL, ml, mean±SD | Zhao 2020 | LNG-IUS: n=25 Desogestrel combined with ethinyl estradiol: n=25 | 90.24±5.37 | 104.26±4.72 | P＜0.05 |
|  | Reduction in MBL, %, mean (SD) | Kaunitz 2010 | LNG-IUS: n=82 Medroxyprogesterone acetate: n=83 | 61.7±41.8 | 11.1±42.5 | P＜0.001 |
| at 6-month | Reduction in MBL, %, mean (SD) | Kaunitz 2010 | LNG-IUS: n=82 Medroxyprogesterone acetate: n=83 | 70.8±88.3 | 21.5±35.8 | P＜0.001 |
|  | MBL, ml, mean±SD | Liu 2015 | LNG-IUS: n=50 Norethisterone: n=50 | 12.67±6.3 | 24.12±8.51 | P＜0.05 |
|  |  | Dong 2021 | LNG-IUS: n=40 Norethisterone: n=40 | 22.28±4.55 | 30.93±6.51 | P＜0.05 |
|  |  | Zhong 2015 | LNG-IUS: n=55 Desogestrel combined with ethinyl estradiol: n=55 | 73.19±11.28 | 81.33±13.87 | Significantly reduced MBL in LNG-IUS group compared with control group. |
|  | MBL, ml, median (range) | Reid 2005 | LNG-IUS: n=25 Mefenamic acid: n=26 | 5 (0–45) | 100 (46–168) | P＜0.001 |
| at 12-month | MBL, ml, mean±SD | Shabaan 2011 | LNG-IUS: n=56 Levonorgestrel combined with ethinyl estradiol: n=56 | 44.4±34.9 | 118.2±75.0 | P＜0.001 |
|  |  | Zhong 2015 | LNG-IUS: n=55 Desogestrel combined with ethinyl estradiol: n=55 | 52.93±10.27 | 65.21±11.07 | Significantly reduced MBL in LNG-IUS group compared with control group. |

**Table S4. Detailed description on scales using to assess health-related quality of life (HRQoL)**

| Scales | Detailed description |
| --- | --- |
| Menorrhagia severity score | Menorrhagia severity score used the condition specific questionnaire. Women completed this questionnaire at baseline and at three-month intervals up to 12 months. The response values ranged from 0 to 5. The responses to all the questions were totaled and divided by the total possible score (which was 42 if all questions were answered). This value was expressed as a percentage, producing a score between 0% (least severe) and 100% (most severe). |
| Short form-questionnaire-36, SF-36 | The SF-36 is a practical and reliable way to obtain important health outcomes data in a variety of settings, measuring eight domains of health: physical functioning, role limitations owing to physical health, bodily pain, general health perceptions, vitality, social functioning, role limitations owing to emotional problems and mental health, with scores ranging from 0 (severely affected) to 100 (not affected). |
| European Quality of Life-5 Dimensions (EQ-5D)/EQ-5D VAS | The EQ-5D is a standardised instrument for use as a measure of health outcome and is widely used in economic evaluations of medical interventions. Applicable to a wide range of health conditions and treatments, it provides a simple descriptive profile (scores ranging from−0.59 (health state worse than death) to 100 (perfect health state)) and also a single-index value for health status measured on a VAS (scores ranging from 0 (worst health state imaginable) to 100 (most perfect health state imaginable)). |
| Sexual activity questionnaire, SAQ | The Sexual Activity Questionnaire (SAQ) was developed as a self-report questionnaire, for use in gynaecological clinical trials, which would be quick to complete and acceptable to the majority of women. Three dimensions of perceptions of sexual activity are measured: pleasure (with scores ranging from 0 (lowest level) to 18 (highest level)), discomfort (scores ranging from 0 (greatest) to 6 (none)) and habit (assessed relative to perceived usual activity as an ordinal response). |
| World health organization quality of life-short form, turkish version, WHOQOL-BREF TR | QOL evaluation was performed according to the World Health Organization Quality of Life-Short Form, Turkish version (WHOQOL-BREF TR), which consists of 26 questions. The participants were asked 7 questions regarding their physical health, 6 about their psychological status, 3 about their social support and 8 relating to their environment. The Turkish version has an additional national item contributing the environmental domain of the scale. Each facet of the WHOQOL-BREF TR is measured using a 5-point Likert scale about the respondents’ feelings over the previous 2 weeks. The range of scores was between 1 and 100, with higher scores indicating better QOL. |
| Health-related quality of life -4, HRQoL-4 | The HRQoL-4 is a brief set of survey based questions designed by The Center for Disease Control to assess HRQoL — defined as “perceived physical and mental health over time.” They include a core set of four questions: (1) Would you say that in general your health is: Excellent, Very good, Good, Fair or Poor? (2) Now thinking about your physical health, which includes physical illness and injury, for how many days during the past 30 days was your physical health not good? (3) Now thinking about your mental health, which includes stress, depression and problems with emotions, for how many days during the past 30 days was your mental health not good? and (4) During the past 30 days, for about how many days did poor physical or mental health keep you from doing your usual activities, such as self-care, work, or recreation? An “unhealthy days” summary measure based on the second and third questions estimates the overall number of recent days when physical or mental health was not good. The complement of this summary measure is “healthy days,” the number of days estimated to be healthy. “Lost days” are the measurement of days that the patient could not go to work or perform her daily activity during the past 30 days, and it is based on the answer for the fourth question. |

**Table S5. Summary results of HRQoL assessed by the short form-questionnaire-36 (SF-36) version 2, the European Quality of Life-5 Dimensions (EQ-5D) and the EQ-5D VAS, the Sexual activity questionnaire (SAQ)**

| **Study** | **Results** | **Follow-up** | **SD** | **95% CI** | | **P-Value** |
| --- | --- | --- | --- | --- | --- | --- |
| Gupta 2015 | SF-36-Physical functioning | at 6-month | 4.7 | 1.2 | 8.1 | p=0.009 |
|  | SF-36-Physical role | at 6-month | 7 | 2.7 | 11.3 | p=0.001 |
|  | SF-36-Emotional role | at 6-month | 7.1 | 2.5 | 11.7 | p=0.002 |
|  | SF-36-Social functioning | at 6-month | 5.6 | 1.4 | 9.8 | p=0.009 |
|  | SF-36-Mental health | at 6-month | 0.6 | –2.5 | 3.8 | p=0.70 |
|  | SF-36-Energy/vitality | at 6-month | 5.9 | 2.0 | 9.7 | p=0.003 |
|  | SF-36-Pain | at 6-month | 10.3 | 6.1 | 14.4 | p<0.001 |
|  | SF-36-General health perception | at 6-month | 2.7 | –0.3 | 5.8 | p=0.08 |
|  | EQ-5D-descriptive system | at 6-month | 0.018 | –0.020 | 0.056 | p=0.36 |
|  | EQ-5D-VAS | at 6-month | 3.1 | –0.4 | 6.5 | p=0.08 |
|  | SAQ-Pleasure | at 6-month | 0.4 | –0.5 | 1.3 | p=0.43 |
|  | SAQ-Discomfort | at 6-month | –0.03 | –0.32 | 0.27 | p=0.87 |
|  | SF-36-Physical functioning | at 6-month | 2.5 | -1.1 | 6.1 | p=0.18 |
|  | SF-36-Physical role | at 12-month | 6.9 | 2.5 | 11.4 | p=0.002 |
|  | SF-36-Emotional role | at 12-month | 3.7 | –0.8 | 8.3 | p=0.11 |
|  | SF-36-Social functioning | at 12-month | 6.2 | 2.0 | 10.5 | p=0.004 |
|  | SF-36-Mental health | at 12-month | 1.8 | –1.5 | 5.2 | p=0.29 |
|  | SF-36-Energy/vitality | at 12-month | 5.8 | 2.1 | 9.5 | p=0.002 |
|  | SF-36-Pain | at 12-month | 9 | 4.4 | 13.7 | p<0.001 |
|  | SF-36-General health perception | at 12-month | 3.7 | 0.4 | 7.0 | p=0.03 |
|  | EQ-5D-descriptive system | at 12-month | 0.026 | –0.020 | 0.071 | p=0.10 |
|  | EQ-5D-VAS | at 12-month | 4.6 | 0.8 | 8.4 | p=0.02 |
|  | SAQ-Pleasure | at 12-month | 0.9 | –0.2 | 1.9 | p=0.43 |
|  | SAQ-Discomfort | at 12-month | –0.20 | –0.54 | 0.14 | p=0.24 |
|  | SF-36-Physical functioning | at 12-month | 1.5 | –2.1 | 5.0 | p=0.42 |
|  | SF-36-Physical role | at 24-month | 3.4 | –0.9 | 7.6 | p=0.12 |
|  | SF-36-Emotional role | at 24-month | 3.3 | –1.2 | 7.8 | p=0.15 |
|  | SF-36-Social functioning | at 24-month | 2.9 | –1.4 | 7.2 | p=0.18 |
|  | SF-36-Mental health | at 24-month | 1.7 | –1.7 | 5.1 | p=0.33 |
|  | SF-36-Energy/vitality | at 24-month | 3.9 | 0.0 | 7.8 | p=0.05 |
|  | SF-36-Pain | at 24-month | 3.9 | –0.6 | 8.5 | p=0.09 |
|  | SF-36-General health perception | at 24-month | 1.3 | –2.3 | 4.9 | p=0.46 |
|  | EQ-5D-descriptive system | at 24-month | 0.007 | –0.031 | 0.045 | p=0.72 |
|  | EQ-5D-VAS | at 24-month | –0.7 | –4.2 | 2.7 | p=0.68 |
|  | SAQ-Pleasure | at 24-month | –0.1 | –1.1 | 0.9 | p=0.83 |
|  | SAQ-Discomfort | at 24-month | 0 | –0.33 | 0.32 | p=0.99 |
|  | SF-36-Physical functioning | 24 month-Overall | 2.7 | 0.0 | 5.4 | p=0.05 |
|  | SF-36-Physical role | 24 month-Overall | 5.9 | 2.6 | 9.1 | p < 0.001 |
|  | SF-36-Emotional role | 24 month-Overall | 4.6 | 1.3 | 8 | p=0.007 |
|  | SF-36-Social functioning | 24 month-Overall | 5.1 | 2.0 | 8.1 | p=0.001 |
|  | SF-36-Mental health | 24 month-Overall | 1.5 | -1.0 | 3.9 | p=0.23 |
|  | SF-36-Energy/vitality | 24 month-Overall | 5.3 | 2.5 | 8.2 | p < 0.001 |
|  | SF-36-Pain | 24 month-Overall | 7.8 | 4.5 | 11.0 | p<0.001 |
|  | SF-36-General health perception | 24 month-Overall | 2.9 | 0.3 | 5.4 | p=0.03 |
|  | EQ-5D-descriptive system | 24 month-Overall | 0.013 | –0.016 | 0.042 | p=0.38 |
|  | EQ-5D-VAS | 24 month-Overall | 2.0 | -0.5 | 4.6 | p=0.12 |
|  | SAQ-Pleasure | 24 month-Overall | 0.4 | -0.3 | 1.1 | p=0.26 |
|  | SAQ-Discomfort | 24 month-Overall | –0.07 | –0.30 | 0.16 | p=0.55 |
|  | SF-36-Physical functioning | 24 month-Overall | 1.6 | –2.7 | 5.9 | p=0.5 |
|  | SF-36-Physical role | at 6-month | 2.7 | –2.1 | 7.5 | p=0.3 |
|  | SF-36-Emotional role | at 6-month | –2.0 | –6.8 | 2.9 | p=0.4 |
|  | SF-36-Social functioning | at 6-month | 2.2 | –2.5 | 6.9 | p=0.4 |
|  | SF-36-Mental health | at 6-month | –1.6 | –5.2 | 2.0 | p=0.4 |
|  | SF-36-Energy/vitality | at 6-month | 2.8 | –1.2 | 6.9 | p=0.2 |
|  | SF-36-Pain | at 6-month | 3.7 | –1.3 | 8.7 | p=0.1 |
|  | SF-36-General health perception | at 6-month | 4.7 | 0.6 | 8.8 | p=0.02 |
|  | EQ-5D-descriptive system | at 6-month | –0.02 | –0.06 | 0.02 | p=0.4 |
|  | EQ-5D-VAS | at 6-month | 0.6 | –3.2 | 4.5 | p=0.8 |
|  | SAQ-Pleasure | at 6-month | –0.4 | –1.7 | 0.9 | p=0.6 |
|  | SAQ-Discomfort | at 6-month | 0.0 | –0.4 | 0.4 | p=0.9 |

Notes: Comparator group including both steroidal medical treatment and non-steroidal medical treatment (mefenamic acid; tranexamic acid; norethisterone; a combined oestrogen–progestogen or progesterone-only oral contraceptive pill (any formulation); medroxyprogesterone acetate injection)

### Table S6. Summary results of HRQoL assessed by WHOQOL-BREF TR

| **Study** | **Results** | **Follow-up** | **LNG-IUS** | | | **Norethisterone** | | | **Tranexamic acid** | | | **P-value** |
| --- | --- | --- | --- | --- | --- | --- | --- | --- | --- | --- | --- | --- |
|  |  |  | mean | sd | total | mean | sd | total | mean | sd | total |  |
| Kiseli 2016 | WHOQOL-BREF TR-Physical domain, Change data | at 6-month | 1.69 | 2.76 | 20 | 1.23 | 3.68 | 22 | 2.21 | 3.84 | 20 | P=0.66 |
|  | WHOQOL-BREF TR-Psychological domain, Change data | at 6-month | 1.17 | 2.93 | 20 | 0.50 | 1.90 | 22 | 1.12 | 3.03 | 20 | P=0.678 |
|  | WHOQOL-BREF TR-Social domain, Change data | at 6-month | –0.73 | 3.42 | 20 | 0.67 | 2.89 | 22 | 0.55 | 3.46 | 20 | P=0.328 |
|  | WHOQOL-BREF TR-Environmental domain, Change data | at 6-month | –0.13 | 2.30 | 20 | 0.03 | 2.50 | 22 | 0.64 | 2.29 | 20 | P=0.543 |
|  | WHOQOL-BREF TR-Environmental domain-TR, Change data | at 6-month | –0.04 | 2.11 | 20 | 0.00 | 2.13 | 22 | 0.36 | 2.34 | 20 | P=0.803 |

### Table S7. Summary results of HRQoL assessed by HRQoL-4

| **Study** | **Results** | **Follow-up** | **LNG-IUS** | | | **Medical treatments** | | | **P-Value** |
| --- | --- | --- | --- | --- | --- | --- | --- | --- | --- |
|  |  |  | Mean | SD | Total (number ) | Mean | SD | Total (number ) |  |
| Shabaan 2011 | HRQoL-4-Physically unhealthy days | at 12-month | 3.7 | 2.0 | 56 | 4.7 | 2.7 | 56 | P=0.186 |
|  | HRQoL-4-Mentally unhealthy days |  | 6.7 | 3.1 | 56 | 4.4 | 1.7 | 56 | P=0.003 |
|  | HRQoL-4-Activity limitation days (lost days) |  | 1.6 | 2.4 | 56 | 6.7 | 2.2 | 56 | P＜0.001 |
|  | HRQoL-4-Self-rated health (≥ very good) |  | Event (number ) | | Total (number ) | Event (number ) | | Total (number ) | P=0.129 |
|  |  |  | 15 | | 56 | 13 | | 56 |  |

### Table S8. Summary results of hemoglobin

| **Follow-up** | **Results** | **Study** | **Sample Size** | **LNG-IUS** | **Medical treatments** | **Conclusion** |
| --- | --- | --- | --- | --- | --- | --- |
| at 6-month | NR | Kiseli 2016 | LNG-IUS: n=20 Norethisterone: n=20 Tranexamic acid: n=22 | NR | NR | LNG-IUS versus norethisterone: P=0.028 LNG-IUS versus tranexamic acid: P<0.001 |
|  | Hemoglobin level, g/dL, mean±SD | Malik 2020 | LNG-IUS: n=38  Norethisterone: n=38 | 10.89±0.3 | 10.82±0.32 | P>0.05 |
|  |  | Kavasoglu 2020 | LNG-IUS: n=97  Norethisterone: n=95 | 11.74±0.96 | 10.97 ± 0.77 | P=0.013 |
| at 12-month | Increase in Hemoglobin, g/L, mean | Endrikat 2009 | LNG-IUS: n=20 Norethindrone acetate combined with ethinyl estradiol: n=19 | 8.6 | 9.6 | P = 0.711 MD, –0.99; 95% CI, –6.43 to 4.45 |
|  | Hemoglobin, g/dL, mean±SD | Shabaan 2011 | LNG-IUS: n=56 Levonorgestrel combined with ethinyl estradiol: n=56 | 11.4±1.0 | 10.1±1.2 | P<0.001 |
|  |  | Kavasoglu 2020 | LNG-IUS: n=97  Norethisterone: n=95 | 12.77±0.56 | 11.35±0.67 | P<0.001 |

**Figure S1. Risk of bias summary**


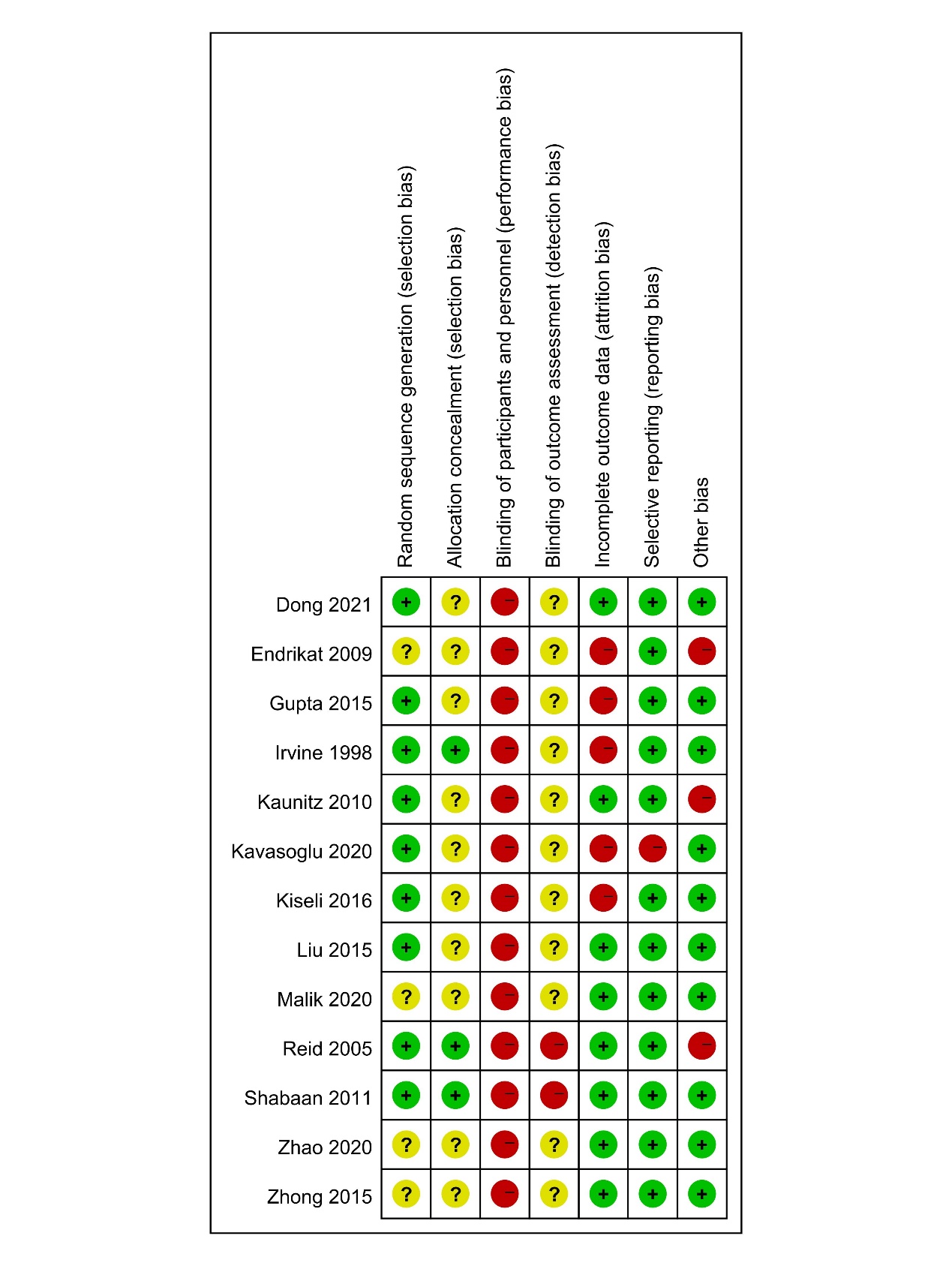


**Figure S2. Forest plots of meta-analysis of LNG-IUS compared with medical treatments for abdominal pain**


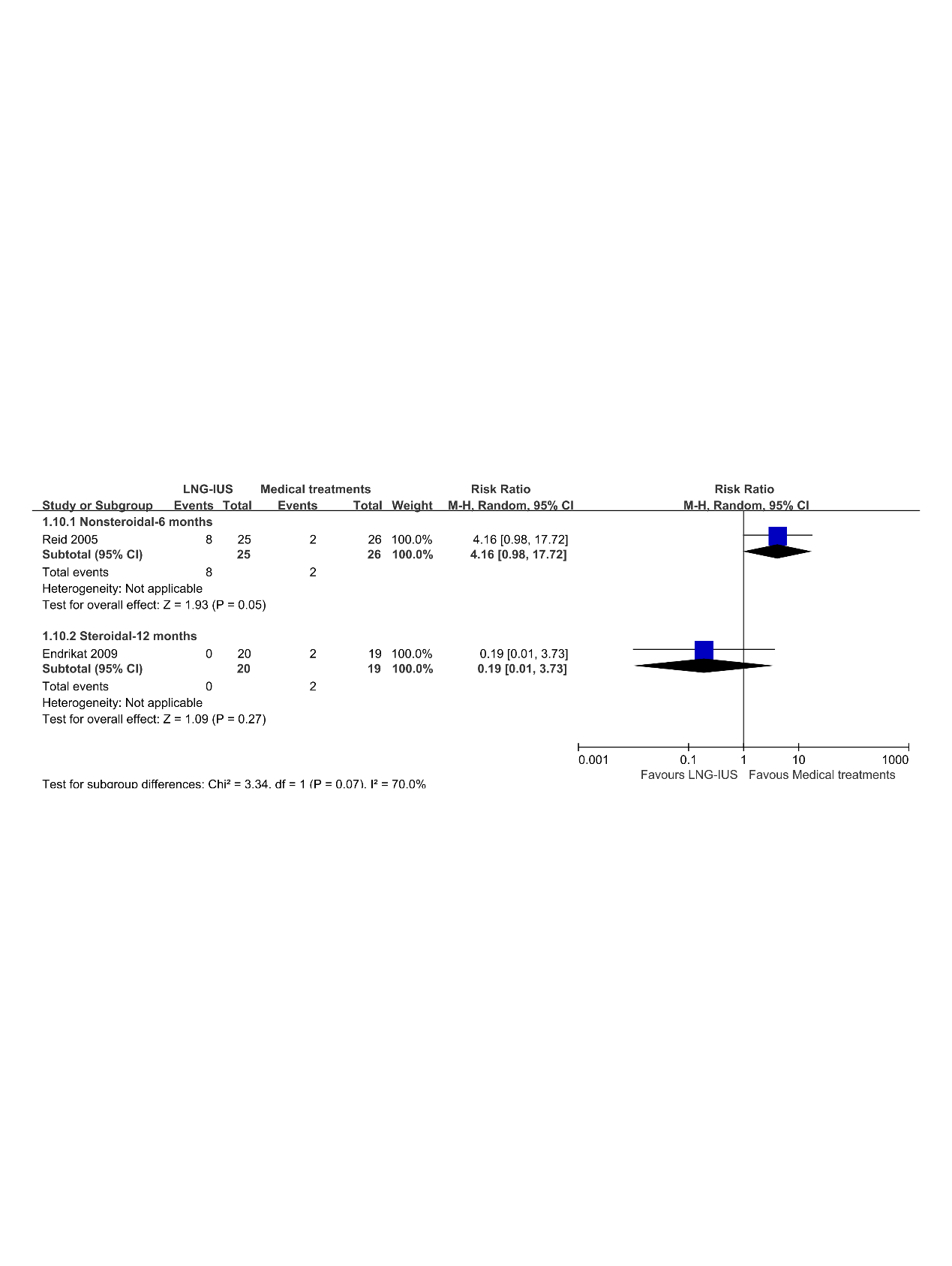


**Figure S3. Forest plots of meta-analysis of LNG-IUS compared with medical treatments for breast tenderness**


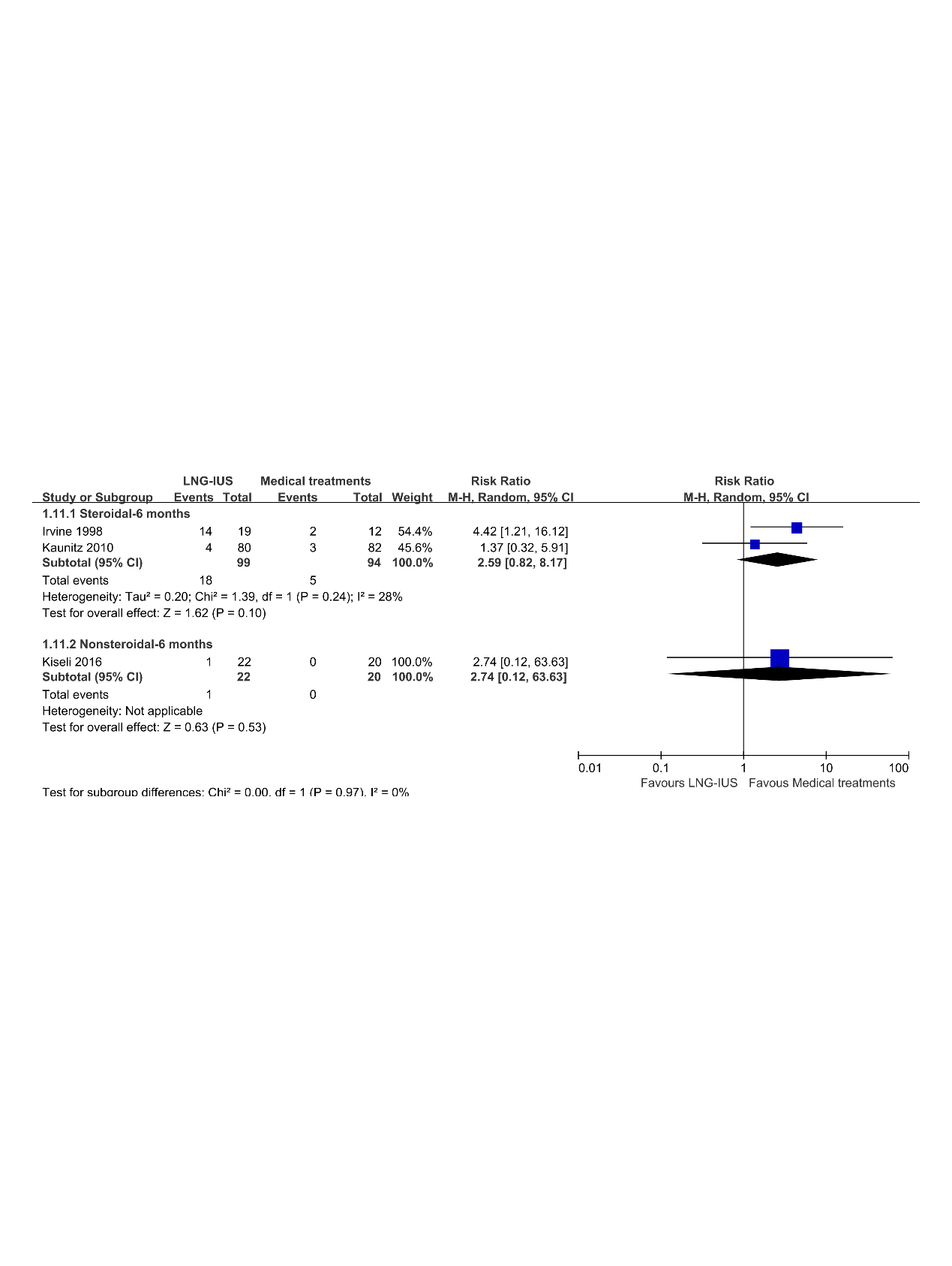


**Figure S4. Forest plots of meta-analysis of LNG-IUS compared with medical treatments for headache**


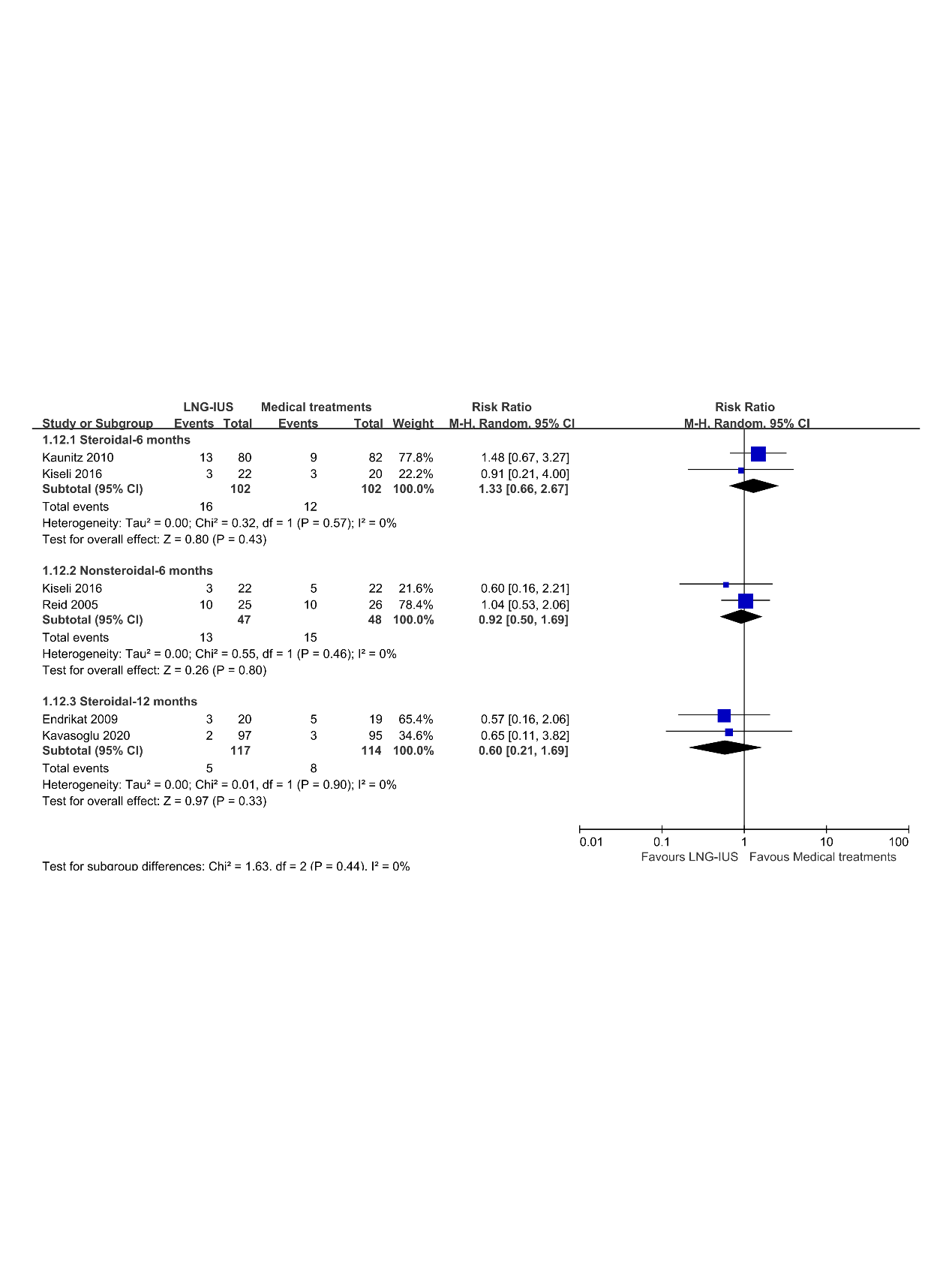


**Figure S5. Forest plots of meta-analysis of LNG-IUS compared with medical treatments for intermenstrual bleeding**


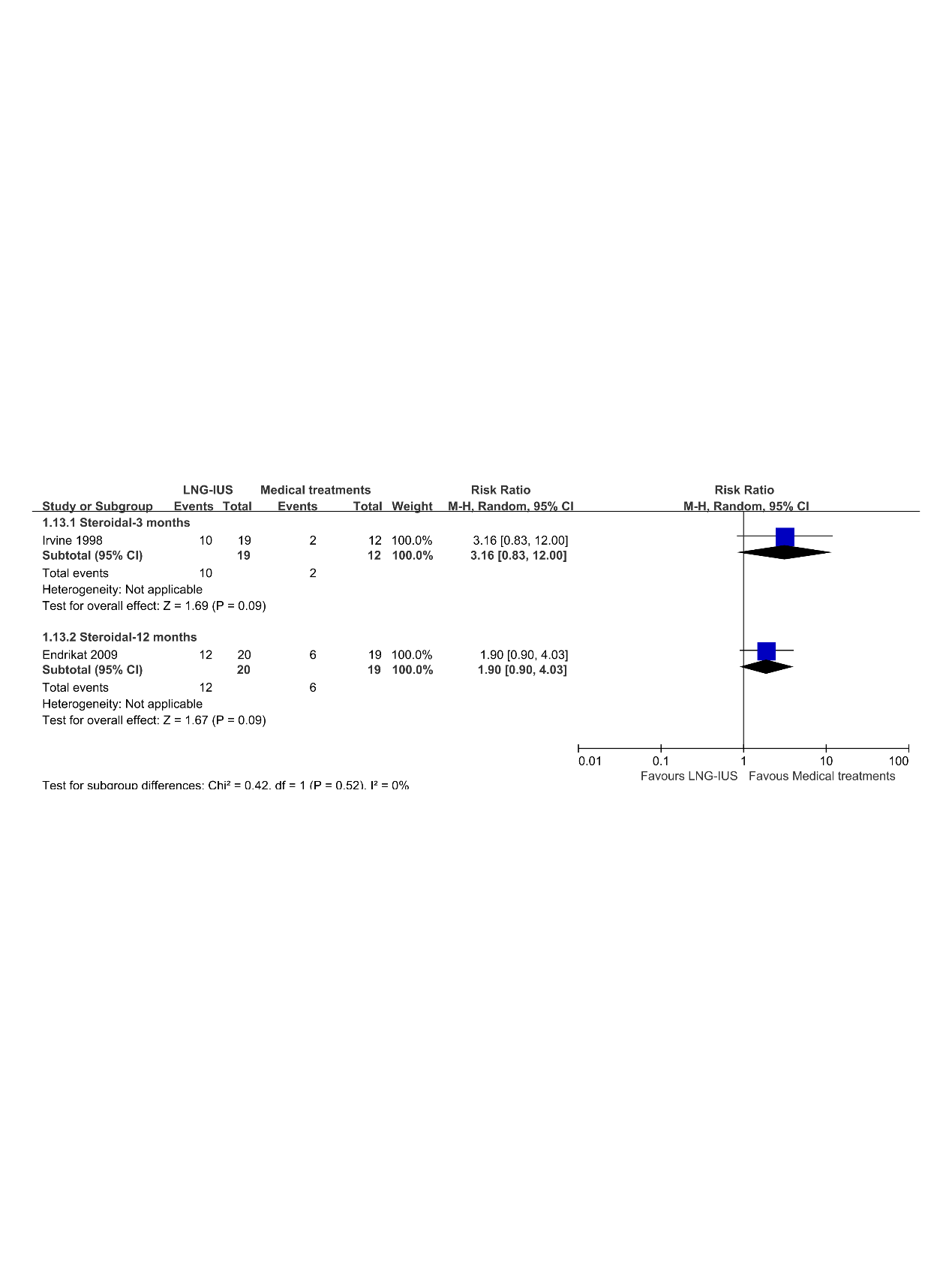


**Figure S6. Forest plots of meta-analysis of LNG-IUS compared with medical treatments for nausea**


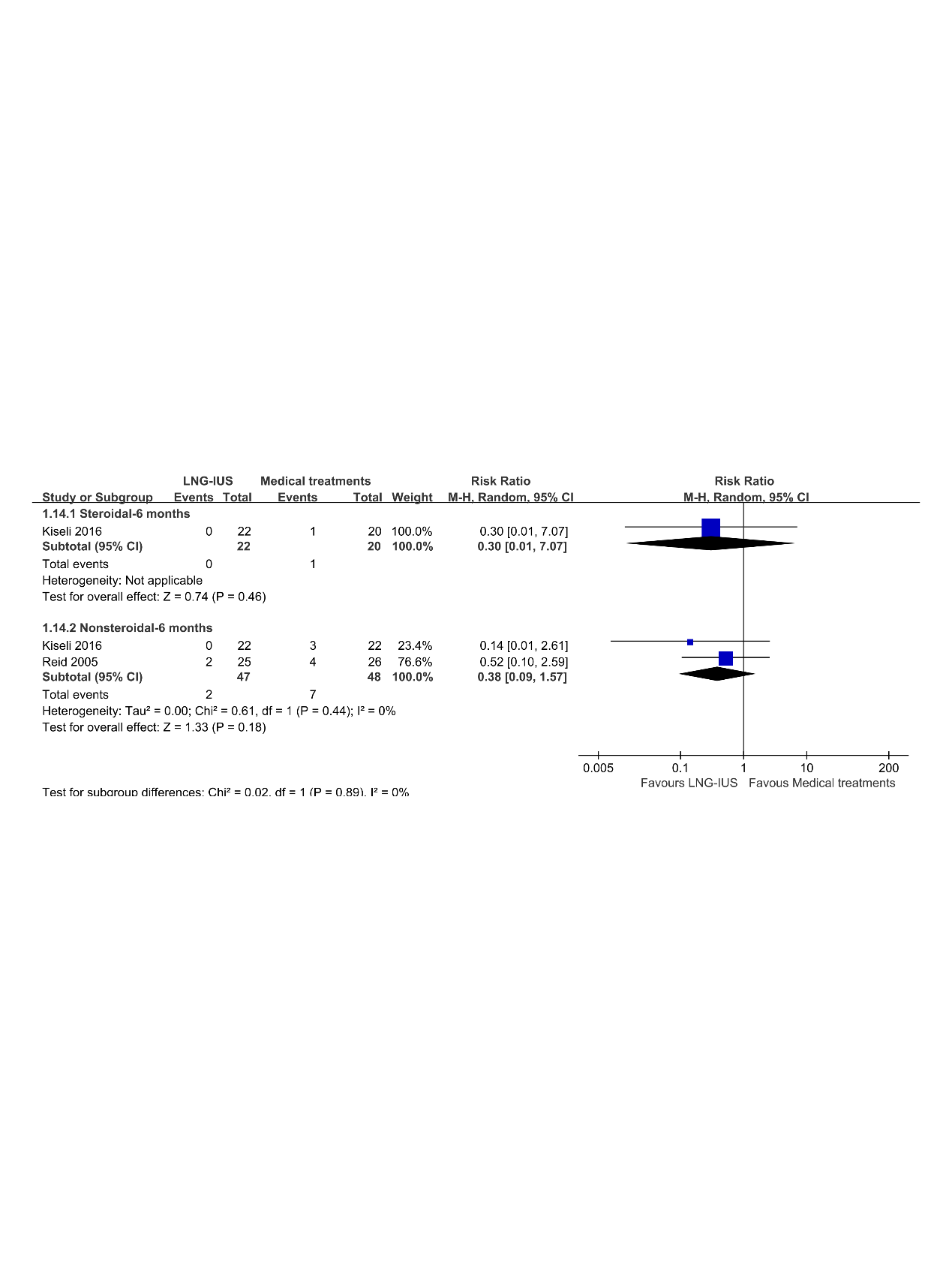


**Figure S7. Forest plots of meta-analysis of LNG-IUS compared with medical treatments for ovarian cyst**

**
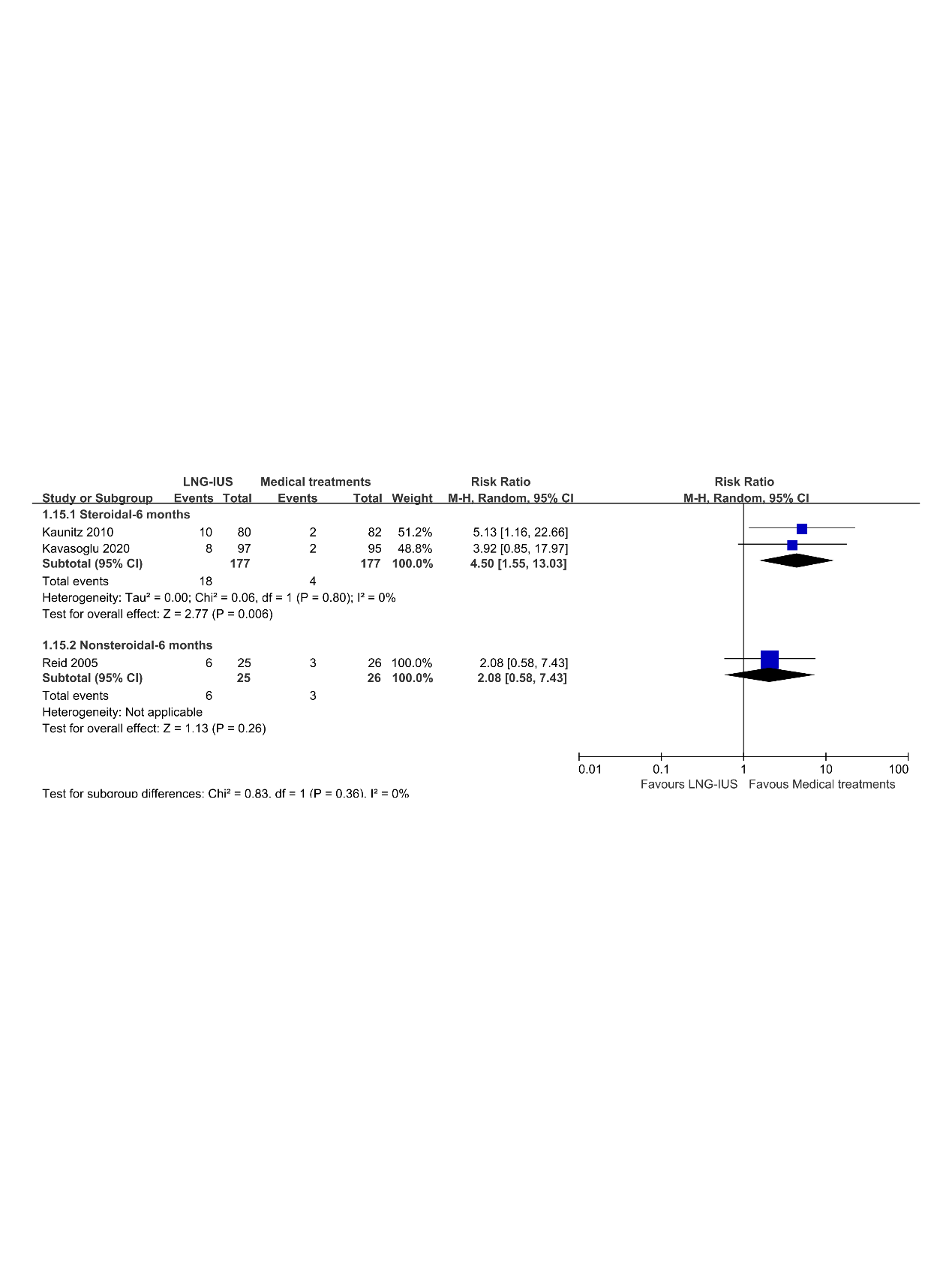
**

**Figure S8. Forest plots of meta-analysis of LNG-IUS compared with medical treatments for weight increase**

**
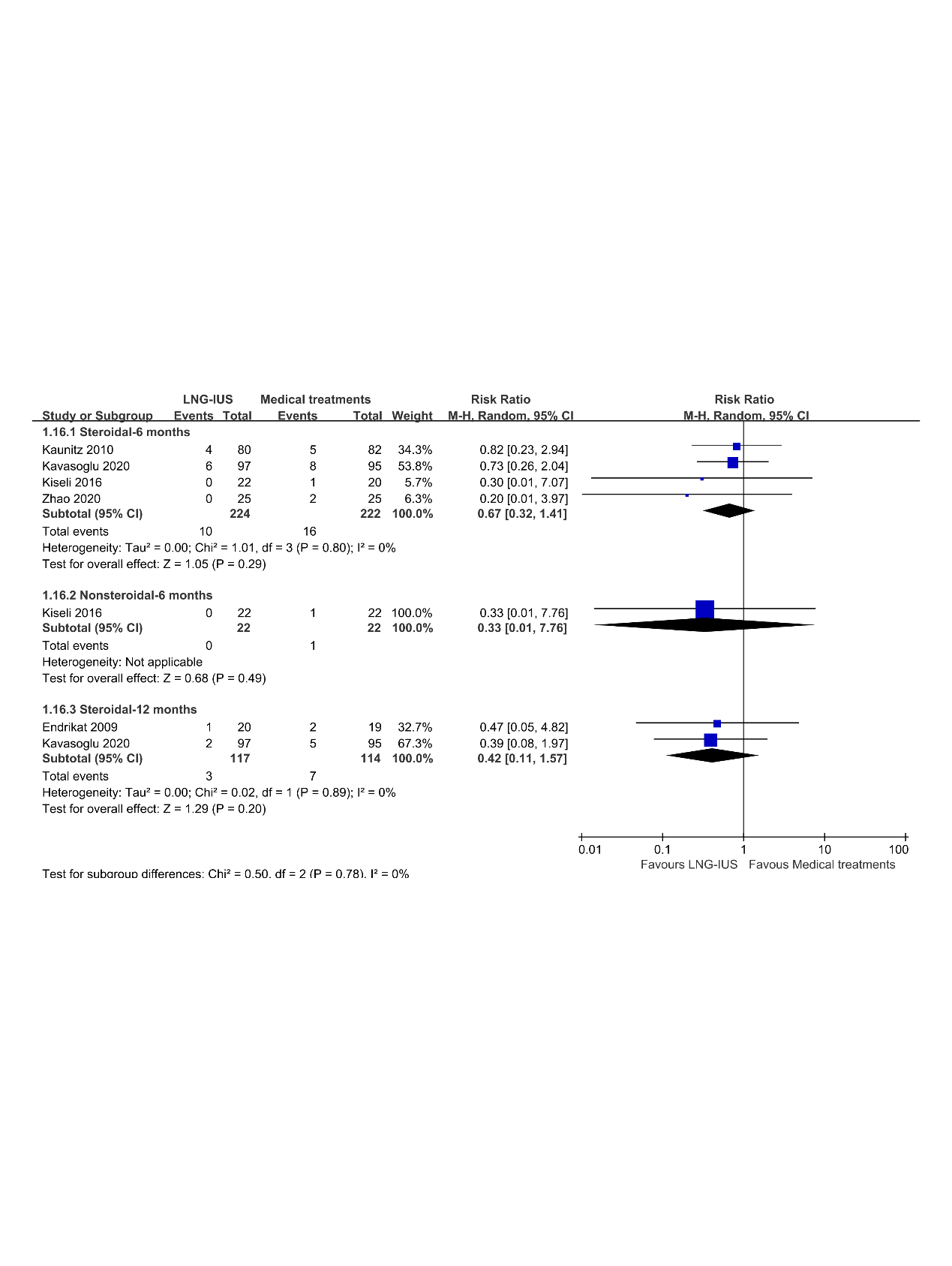
**
